# Psychological Detachment Mediating the Daily Relationship between Workload and Marital Satisfaction

**DOI:** 10.3389/fpsyg.2016.02036

**Published:** 2017-01-04

**Authors:** Lynn Germeys, Sara De Gieter

**Affiliations:** Faculty of Psychology and Educational Sciences, Work and Organizational Psychology, Vrije Universiteit BrusselBrussels, Belgium

**Keywords:** workload, psychological detachment, marital satisfaction, work-home segmentation preference, diary study

## Abstract

Scholars already demonstrated that psychologically detaching from work after workhours can diminish or avoid the negative effects of job demands on employees' well-being. In this study, we examined a curvilinear relationship between workload and psychological detachment. Moreover, we investigated the moderating influence of an employee's work-home segmentation preference on the relation between detachment and marital satisfaction. In addition, we applied and extended the stressor-detachment model by examining detachment as a mediator of the relation between workload and marital satisfaction. A total of 136 employees participated in our daily diary survey study during 10 consecutive working days. The results of the Bayesian 2-level path analyses revealed a negative linear and curvilinear relationship between workload and psychological detachment on a daily basis. Daily detachment positively related to marital satisfaction, with one's preference to segment work from home reinforcing this relationship. Moreover, psychological detachment fully mediated the daily relationship between workload and marital satisfaction. Implications for practice and suggestions for future research are discussed.

## Introduction

Since the rise of the number of dual-earner couples, research examining the interaction between the work and home domain increased. By now it is well established that work experiences exert influences outside the work domain (e.g., Martinez-Corts et al., 2015). Paid work takes up a considerable amount of time in individuals' lives, during which they face different job demands (Landy and Conte, 2016). Workload is one important demand—referring to the amount of work employees have to handle within their time at work (Jex, 1998)—that results in mainly negative outcomes for the employee at work (e.g., exhaustion at work; Bakker et al., 2003) and at home (e.g., work-family conflict; Ilies et al., 2007).

According to the stressor-detachment model, an individual can obviate negative home outcomes resulting from job demands such as workload by recovering from work (Sonnentag, 2010). This can be done by cognitively and physically restraining from work-related activities and experiences during one's non-working time (i.e., psychologically detaching from work after workhours; Sonnentag and Bayer, 2005). In turn this capacity to “mentally switch off” from work has benefits for the individual, such as higher life satisfaction and less psychological strain (Sonnentag, 2012).

With this study we aim to fill current research gaps and as such contribute to the literature by extending and advancing the theoretical understanding of the current stressor-detachment model (Sonnentag, 2010; Sonnentag and Fritz, 2015). Previous between-person (Sonnentag et al., 2010b; Safstrom and Hartig, 2013; Potok and Littman-Ovadia, 2014) as well as within-person studies (Sonnentag and Bayer, 2005) provided support for a linear negative relationship between workload and psychological detachment. High levels of workload seem to—linearly—hinder an employee's ability to psychologically detach from work during the evening (Sonnentag and Bayer, 2005; Sonnentag and Kruel, 2006). In this study we respond to recent calls to explore possible non-linear relationships instead of assuming linear ones (Busse et al., 2016) and hypothesize that not only high but also low levels of workload (i.e., overload and underload) can interfere with the capacity to detach from work. As such, we aim to extend our current knowledge on the daily relationship between workload and psychological detachment, by examining the existence of a curvilinear relationship.

In addition, previous between-person (Moreno-Jiménez et al., 2009b) and within-person studies (e.g., Demerouti et al., 2012) almost exclusively examined health outcomes of detachment. By now it is well documented that psychological detachment relates negatively with strain and positively with well-being outcomes (Sonnentag and Fritz, 2015). However, the influence of detachment on relational outcomes remains unknown (e.g., no reference to relational outcomes such as marital satisfaction in the meta-analysis of Sonnentag and Fritz, 2015). We aim to add to the understanding of consequences of psychological detachment by focusing on marital satisfaction (i.e., relational outcome). Furthermore, research examining buffering or intensifying influences (i.e., moderators) on the link between detachment and outcome variables is limited to non-existing (Sonnentag and Fritz, 2015). To fill this void, we will examine whether employee's work-home segmentation preference—the stable preference to either segment work and private life or to integrate both life domains to a strong extend (Kreiner, 2006)—reinforces the daily relationship between detachment and marital satisfaction. The original stressor-detachment model postulated a moderating as well as a mediating role of detachment in the relation between job stressors and well-being outcomes (Sonnentag, 2010). So far, previous diary studies mainly focused on the moderating role (e.g., Sonnentag et al., 2010a) and—to our knowledge—only one study examined the mediating role of detachment (ten Brummelhuis and Bakker, 2012). We aim to extend the literature by examining whether detachment mediates the daily negative spillover from workload to an employee's marital satisfaction.

Scholars often used cross-sectional research designs to examine the stressor-detachment model (e.g., Sonnentag et al., 2010a), whereas recent empirical studies support a dynamic view on detachment (e.g., Demerouti et al., 2012). Sonnentag and Fritz (2015) called for more research to examine the short-term dynamics (i.e., within one workday) of the stressor-detachment model. In line, we performed a daily diary survey study, which allows us to capture the day-to-day variation.

Besides theoretically advancing the stressor-detachment model literature, we aim to formulate practical implications to help dual-earner couples in the juggle between their work and private life.

### Stressor-detachment model

Nowadays employees work in highly competitive and stressful organizational settings where they encounter many job demands (e.g., workload, emotional demands, cognitive demands; Landy and Conte, 2016). These stressful work situations affect an employee's well-being by resulting in strain and fatigue symptoms (e.g., Bakker et al., 2003). To alleviate the negative consequences of experiencing high levels of job demands, an employee needs to recover from work (Sonnentag et al., 2010a). One strategy to recover from stressful work situations is by psychologically detaching from work. Mentally and physically restraining from work-related activities and experiences at home allows an employee to cease further taxation of resources (e.g., mood, time, energy) and provides opportunities to replenish drained resources (Sonnentag, 2010).

The stressor-detachment model is a theoretical framework that explains the moderating and/or mediating role of detachment in the relationship between job-induced stress and strain outcomes that stem from job stressors experienced at work (Sonnentag and Fritz, 2007a; Sonnentag, 2010). Triggered by the stressor-detachment model, scholars recently started paying more attention to detachment's mediating role instead of the predominantly examined moderating role of detachment (Sonnentag and Fritz, 2015). Here, we will conceptualize detachment as a possible mediator in the relationship between workload and marital satisfaction. Demanding job characteristics such as workload consume employees' personal resources (e.g., time, energy), often evoke negative emotions and keep them preoccupied with work, hindering them to detach (Sonnentag and Bayer, 2005; Bakker and Demerouti, 2007). Yet, research on recovery from work clearly shows that being able to detach from work and forget about the demanding job circumstances increases employees' well-being and reduces strain (Sonnentag and Bayer, 2005). Linking both findings suggests that psychological detachment acts as a mediator and as such explains how and why daily job stressors are related to strain. Moreover, there are also methodological reasons to approach detachment as a mediator. Previous studies only found very small increases in explained variance by adding the moderation by detachment (e.g., Sonnentag et al., 2010a). In addition, previous studies reported high correlations between job stressors and the ability to detach (e.g., Moreno-Jiménez et al., 2009b)—directing at its possible mediating role—, whereas this high correlation is not a prerequisite and might even be detrimental for detachment's moderating role. In contrast to ample research examining the moderating role (e.g., Moreno-Jiménez et al., 2009a), we will advance the stressor-detachment model by performing an empirical test and providing evidence for the existence of the proposed mediating influence of detachment. Moreover, we theoretically advance the stressor-detachment model by extending the model by including moderators (i.e., work-home segmentation preference). In addition, unraveling psychological underlying processes (i.e., identifying a mediator) is especially relevant to develop intervention programs to alleviate the negative influence of job stressors on an employee's well-being (Baron and Kenny, 1986).

### Curvilinear relationship between workload and detachment

Job demands—such as workload—are conceptualized as work conditions that put a burden on an employee's capacities (Bakker and Demerouti, 2007). Recently, scholars who examined the job demands-resources theory differentiated job demands into challenge (e.g., workload) and hindrance (e.g., role ambiguity) demands (LePine et al., 2005). Challenge demands deplete and simultaneously stimulate energy whereas hindrance demands solely deplete energy (Van den Broeck et al., 2010). The dual potential to motivate employees to achieve personal growth and achieve goals as well as to drain an employee's energy suggests a curvilinear relationship between challenge demands and research outcomes (LePine et al., 2005). In different research areas, a non-linear inverted U-shaped relationship is framed in the light of the Yerkes-Dodson law suggesting an optimal mid-range level of arousal, with both extremes of the curve (i.e., very low and very high) leading to less favorable outcomes (Yerkes and Dodson, 1908). In addition, activation theory suggests that people who encounter very low levels of activation at work will be apathetic, increases in activation will energize employees, whereas further increases will drain resources and elicit feelings of inability to cope with the activation (Gardner, 1986; Gardner and Cummings, 1988).

Scholars already found support for a curvilinear relationship between workload and other outcome variables, such as physical health (i.e., cross-sectional study; Karanika-Murray et al., 2009) and task performance (i.e., diary study; Hofmans et al., 2015). In line with the Yerkes-Dodson law (Yerkes and Dodson, 1908) and activation theory (Gardner, 1986; Gardner and Cummings, 1988), scholars found that high, low and moderate levels of workload were in decreasing order related to negative health outcomes (Shultz et al., 2010). Stated differently, whereas the former results in stress due to overload, the second results in boredom due to underload and the latter displays an optimal fit between the work environment and an employee's capacities. Yet other scholars found that overload as well as underload arouse feelings of stress (Gardner, 1986; Fisher, 1991; Richter et al., 2008). Paradoxically, employees who encounter stress are most in need of detachment to stay energized, healthy and engaged, yet, the stressful circumstances make it hard for them to detach from work (Sluiter et al., 2003; Sonnentag et al., 2010a). Combining the abovementioned findings, having too little or too much work to handle is likely to impede with an employee's ability to detach on a daily basis. Underload (i.e., low levels of workload) can hamper the psychological detachment from work as employees feel apathetic, under-stimulated, frustrated and stressed, whereas overload (i.e., high levels of workload) can hamper detachment as employees feel overwhelmed, unable to cope with the stressor, exhausted and stressed (Gardner, 1986; Gardner and Cummings, 1988; Fisher, 1991; Zivnuska et al., 2002; Richter et al., 2008). The optimal mid-range of workload is demanding but workable and as such does not evoke feelings of inability to cope with the workload nor stress reactions (Yerkes and Dodson, 1908; Gardner, 1986; Gardner and Cummings, 1988). In conclusion, we suggest a curvilinear, inverted U-shaped relationship between workload and detachment, and hypothesize that:

*Hypothesis 1: Employees' daily workload is negatively related to their daily ability to psychologically detach from work through a curvilinear/inverted U-shaped function*.

### Linear positive relationship between detachment and marital satisfaction

Many scholars found support for a positive daily relationship between psychological detachment and health outcomes on one hand (e.g., more vigorous and less exhausted; Demerouti et al., 2012) and home outcomes on the other hand (e.g., less work-family conflict; Sanz-Vergel et al., 2011). However, studies linking employees' ability to detach from work to relational outcomes is limited or even non-existing (e.g., no reference to relational outcomes in the meta-analysis of Sonnentag and Fritz, 2015). Detaching from work-related activities and experiences at home allows an employee to cease further (threat of) loss of resources and provide opportunities to replenish drained resources (Sonnentag and Fritz, 2007a). According to conservation of resources theory (Hobfoll, 1988, 1989) individuals strive to preserve and protect already acquired resources, and furthermore gain additional resources. Moreover, individuals who actually lose or face the threat of losing resources are more prone to experience strain. As a consequence, employees who are unable to detach will experience a continuing taxation of resources and consequently negative outcomes, whereas an employee who is able to detach can replenish drained resources and as such alleviate negative outcomes (Hobfoll, 1989; Sonnentag and Fritz, 2015). Prior scholars found that in order to maintain positive relational functioning, communication and behavior, spouses need to rely on and invest resources (e.g., self-control; Neff and Karney, 2009; Randall and Bodenmann, 2009). Hence, the inability to detach from work will further tax the limited pool of resources and impair marital satisfaction, whereas detaching from work will replenish resources which will contribute to and benefit feelings of marital satisfaction. As such, we hypothesize that:

*Hypothesis 2: Employees' daily ability to psychologically detach from work is positively related to marital satisfaction*.

### Moderating role of work-Home segmentation preference on the daily relation between detachment and marital satisfaction

Individuals who combine work and family responsibilities can either prefer to segment the work and home domain or to integrate both domains (Kreiner, 2006). The former refers to an employee's preference to maintain impermeable boundaries between work and home (i.e., keep both domains separated), whereas the latter refers to permeable work and home boundaries (i.e., both domains are blended). According to boundary theory, individuals can experience a violation of their work-home boundaries when their boundary preference does not align with how their boundaries are treated (Kreiner et al., 2009). Experiencing work-home boundary violation can result in negative home outcomes (i.e., work-home conflict; Kreiner et al., 2009). According to person-environment fit theories, individuals will experience more positive outcomes if they act congruent with their preference whereas a mismatch between individuals' acts and preferences will result in more negative outcomes (Kristof-Brown et al., 2005). As such, we argue that on days during which employees are not able to detach—that is, not able to keep work separated from the home domain—, the negative influence on their perceived marital satisfaction will be even stronger for those employees who generally prefer to segment work and home domains. Contrary, we assume that one's preference to segment work from home will strengthen the positive influence of detachment on marital satisfaction, since being able to psychologically detach from work aligns with an individual's preference to segment work and home boundaries. As such, we hypothesize that:

*Hypothesis 3: An employee's work-home segmentation preference moderates the daily relationship between psychological detachment from work and marital satisfaction, such that detachment will lead to more marital satisfaction when the employee's preference to segment work and family life is high*.

### Mediating role of detachment in the linear relation between workload and marital satisfaction

Meta-analytic findings suggest associations between the experience of workload at work and negative outcomes (e.g., psychological and physical well-being; Bowlinga et al., 2015). In addition, recent diary studies found that workload negatively related to an employee's marital life (Story and Repetti, 2006; Lavee and Ben-Ari, 2007). Nevertheless, these studies mainly focused on constructs related to marital satisfaction. However, marital satisfaction in itself is an important outcome as it predicts aspects of well-being (Proulx et al., 2007). According to the conservation of resources theory, at low levels of workload employees do not need to address their limited pool of resources (Hobfoll, 1989). Consequently, one could argue that this will positively impact an employee's marital satisfaction as they can employ these resources at home. However, moderate and high levels of workload require an employee to use resources in order to handle the workload (Hobfoll, 1989). As such, with increasing levels of workload and the taxation of resources that goes with it, we assume an employee's feelings of marital satisfaction will go down as the consumed resources are no longer available in the home domain to invest in one's relationship. In other words, we hypothesize that daily workload will interfere with marital satisfaction. However, previous research found that workload only indirectly—via mood—influenced one's marital life (i.e., dyadic closeness; Lavee and Ben-Ari, 2007). In a similar vein, we assume that the influence of daily workload on marital satisfaction operates through psychological detachment. More specifically, when an employee experiences low or high levels of workload this will intervene with his/her ability to detach (i.e., hypothesis 1). Not being able to detach will result in a prolonged influence of the job demands at home and as such in a decrease in marital satisfaction (i.e., hypothesis 2). One way to intervene in the negative spillover from work experiences to marital satisfaction, is by recharging one's batteries, i.e., not thinking nor working on job-related matters. As such, we hypothesize that:

*Hypothesis 4: Employees' daily ability to psychologically detach from work mediates the negative relationship between workload and marital satisfaction*.

**Figure 1:**
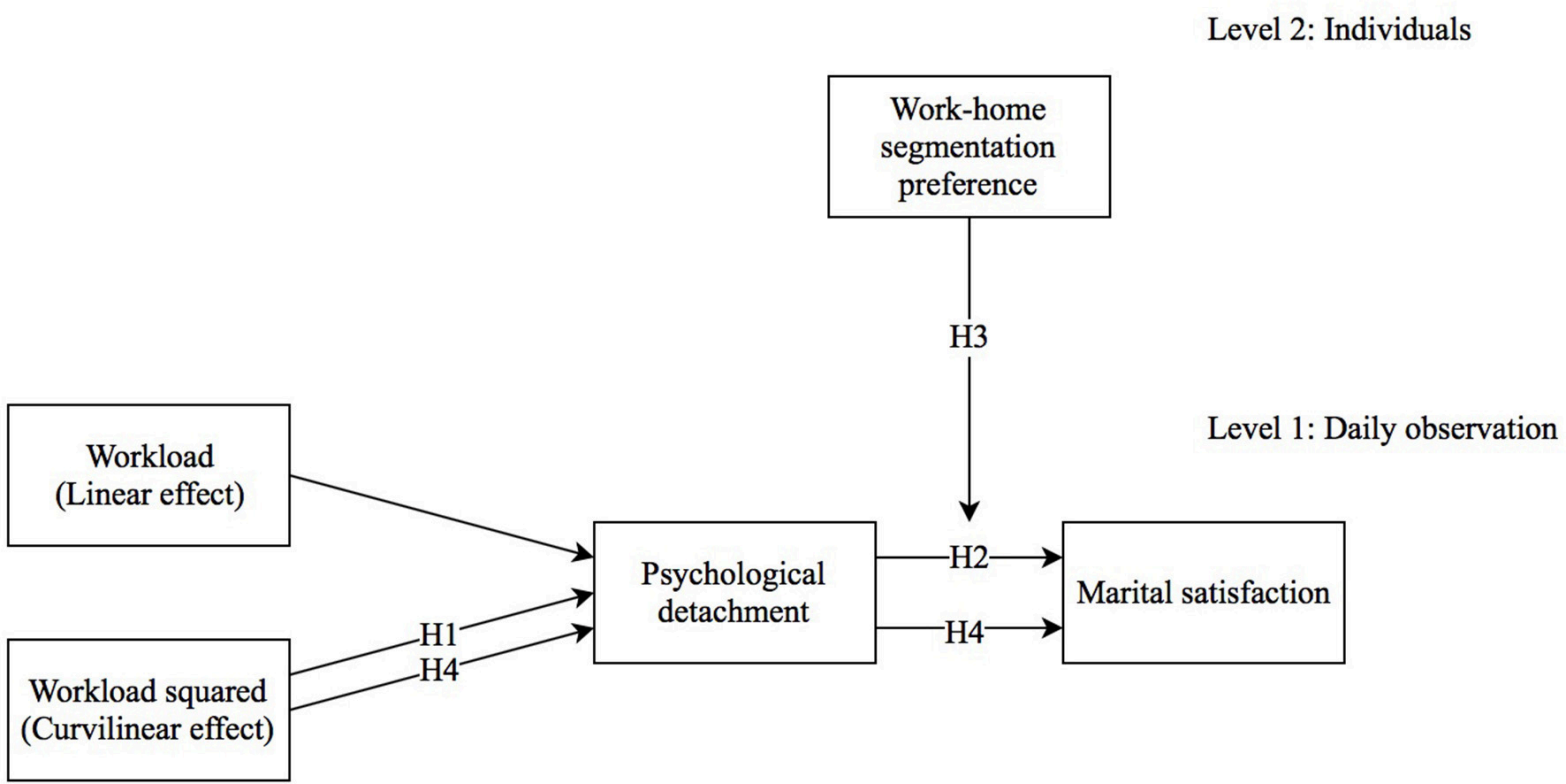
**The study hypotheses**.

The study hypotheses are graphically depicted in Figure 1.

## Methods

### Procedure

We contacted individual Belgian employees from different sectors (e.g., healthcare, banking, education, and justice) by means of a convenience sampling approach (i.e., using the researchers' personal network and word-of-mouth communication). Respondents could not be self-employed, had to work at least 50% and had to be part of a dual-earner couple—cohabiting partners in a romantic relationship (either married or unmarried) of which both partners work at least part-time as paid employees in a variety of sectors—to participate in this study. During a personal conversation with each respondent, we explained the purpose of the study, stressing the discretionary nature of participation, the possibility to withdraw from the study at any time, and the confidential treatment of the data. In addition, each respondent received written information about the study and a personal code. This code assured respondents' anonymity and allowed us to match their general and diary surveys afterwards. Each respondent also received an envelope with this personal code, which we individually collected after completion. All respondents indicated their willingness to participate by signing an informed consent. No incentives were provided for participation in the research. The university's ethics committee granted ethical approval for the study (reference number ECHW_045).

We opted for this daily survey design as it reduces the retrospective bias of more traditional survey studies (Reis and Gable, 2000) and allowed us to account for the situational and temporal context when studying feelings, cognitions and behaviors (Reis and Gable, 2000). Moreover, we opted for a study period of two workweeks, since prior studies found that during this time period it should be possible to capture respondents' life representatively (Reis and Wheeler, 1991). We asked our respondents to complete a one-time general survey 1 week prior to completing the daily diary study for 10 consecutive working days (i.e., not during weekends) starting on a Monday. All respondents had workweeks of 5 consecutive days, namely from Monday till Friday. Each workday, respondents needed to fill out the same questionnaire to rate the level of workload, psychological detachment and marital satisfaction experienced that day. We instructed our respondents to not look back to their answers of the previous day(s) and stressed the fact that there were no right nor wrong answers. To lower the burden of the respondents, we only collected data once a day, right before bedtime. For their convenience, we instructed them to keep their paper diary booklet on their nightstand at home. We emphasized that they were not required to fill out the daily diary survey on days they did not work, for example due to part-time working or illness. These days were treated as missings. We only included data of part-time and full-time employees who completed more than three out of ten daily diary surveys in a timely manner (i.e., completed on the requested day according to their self-reported time stamps) to minimize the effect of recollection bias. Overall we had 1144 observations (out of 1360, compliance rate = 84.12%).

### Participants

A total of 136 Belgian employees working in different sectors participated in our study. About half of the respondents were women (48%) with an average age of 41.04 years (*SD* = 10.72, range: 21–59 years). All respondents obtained at least a secondary school degree and the majority were employed as white collar worker (90%). They exerted their current function on average for 11.46 years (*SD* = 9.85) and most of them worked full-time (79%; see Appendix A in Supplementary Material for the results of the multi-group comparison test between full-time and part-time workers). The majority of the respondents had at least one child (79%).

Note that due to the multilevel nature of the data, the unit of analysis equals “daily diary survey entries” rather than “respondents” (Conway and Briner, 2002) for the level 1 hypotheses (i.e., hypotheses 1, 2, and 4). As a result, the sample size contains 1144 observations (136 respondents x a maximum of 10 daily diary survey entries), or an average of 8.41 completed daily diary surveys per respondent. For the cross-level hypothesis (i.e., hypothesis 3), the unit of analysis equals “respondents,” resulting in 136 respondents. In this respect, Maas and Hox (2005) found that level 2 sample sizes exceeding 30 (i.e., 136 in our study) are sufficiently large to produce unbiased estimates and accurate estimations of standard errors and fixed effects.

### Measures

All items were rated on a five-point Likert scale ranging from “*Completely not agree*” (1) to “*Completely agree*” (5).

#### General survey measures

We used the general survey to collect demographic information and work-home segmentation preference. *Work-home segmentation preference* was measured using the four-item scale of Kreiner (2006) including items such as “I don't like to have to think about work while I'm at home.” We operationalized this preference to segment or integrate work and home as a stable trait, in accordance with the existing literature (Kreiner et al., 2009) and therefore assume that this preference will not change over the course of two workweeks. We translated the original English items (i.e., developed in the United States) to Dutch, using the back-translation procedure (Brislin, 1980). Two bi-lingual (Dutch–English) translators who are familiar with the Belgian culture and the research topic independently translated the items. Afterwards two other bi-lingual translators checked the translations on inconsistencies, discussed these and resolved any deviation between the original and translated items. In addition, these translators checked for cultural sensitivities, to avoid cultural inappropriate translations and the similarity in meaning between the original and translated items. Afterwards, before administrating the translated items, we field-tested the wording and meaning of the items with two respondents who were not familiar with the research topic. The alpha reliability coefficient of this scale with the current sample was 0.70.

#### Daily survey (before bedtime)

*Workload* was measured with three items based on the Dutch version (Furda, 1995) of the Job Content Instrument (Karasek, 1985). We slightly modified the existing scale (by adding “today” to the item; for a similar approach see for example Ilies et al., 2007) to capture the daily time frame. The scale includes items such as “Today I had to work fast.” The within-person omega reliability coefficient of this scale with the current sample was 0.88.

*Psychological detachment* was measured with the Dutch translation (Geurts et al., 2009) of the four-item Recovery Experience Scale of Sonnentag and Fritz (2007b), including items such as “After work, I could distance myself from my work.” The within-person omega reliability coefficient of this scale with the current sample was 0.88.

*Marital satisfaction* was measured—in line with prior research (Buunk and Bakker, 1997)—with two Dutch items from the Relational Interaction Satisfaction Scale of Buunk (1990). We slightly modified the existing items to capture the daily time frame (e.g., adding “today” to the item). Although two-item measures are rarely used in traditional (i.e., cross-sectional) designs, in diary studies single- and two-item measures have a considerable history especially for concrete constructs. However, the potential downsides of the use of a shortened scale are the risks of low reliability and the inappropriateness to measure multi-dimensional constructs (Smith et al., 2000). Nevertheless, according to Anderson et al. (2009), marital satisfaction is a concrete, homogeneous and unidimensional construct. We selected the two items that correlated the highest with other relational satisfaction scales (Buunk and Bakker, 1997). A sample item is: “Today, I felt happy with my partner.” The within-person omega reliability coefficient of this scale with the current sample was 0.94.

### Data analysis

Given the nested structure of our data (i.e., working days nested within employees), we performed two-level path analyses using Mplus version 7.3 (Muthén and Muthén, 2012), in which we separated within- and between-components (Preacher et al., 2010). Prior to testing our hypotheses, we conducted a multilevel confirmatory factor analysis (CFA) to examine the discriminant validity of our research variables. Prior to specifying the within-person part of the two-level path model, we person-mean centered the level 1 predictor variables (i.e., workload and squared workload) at an employee's individual mean to eliminate between-person variance (Hofmann et al., 2000). As such, for the hypotheses pertaining to the within-person level (i.e., hypotheses 1, 2, and 4) the predictor variables only contain within-person variability. Note that before squaring workload, we first person-mean centered this variable due to the high risk of multicollinearity otherwise. In addition, it is recommended to include the first order regression term (i.e., linear) as well, when examining a higher order regression (i.e., quadratic). Prior to specifying the moderated two-level path model, we grand-mean centered the cross-level moderator (i.e., work-home segmentation preference) at the overall mean.

First, we examined the intercept-only model to estimate the amount of variance attributable to the person (i.e., level 2) and day (i.e., level 1) level of the model. Next, we modeled relationships among within-person variables (workload, squared workload, detachment, and marital satisfaction) at level 1 by defining random slopes. We compared the balance between the number of parameters (i.e., model complexity) and the fit of the model to the data (i.e., Bayesian Information Criterion or BIC) of a full and a partial mediation model. According to the BIC values, the full mediation model yielded a superior fit to the data (BIC_full mediation_ = 5092.61 < BIC_partial mediation_ = 5097.96; Aiken and West, 1991). Consequently, we will rely on the full mediation model when discussing the results. We tested the cross-level moderation by examining the influence of work-home segmentation preference on the strength of the level 1 relationship (i.e., between detachment and marital satisfaction; Cohen et al., 2003).

To simultaneously test the non-linear mediation and cross-level moderation we rely on Bayesian two-level path modeling. The reason to opt for Bayesian estimation is threefold: (1) it can handle complicated models, (2) it can handle missing data (e.g., due to working part-time) well by using all observations to estimate parameters without imputing data, and (3) it is suited for hierarchical non-normal distributions, which is traditionally the case when testing multilevel mediation by the use of the product-of-coefficient approach. Bayesian analysis deviates from traditional frequentist analyses as it provides a posterior distribution (i.e., probability distribution of each parameter) and credibility intervals (i.e., 95% most credible parameter values) instead of a *p*-value and/or confidence intervals. We will rely on the credibility intervals (CI) to determine whether a parameter value is credible (Kruschke et al., 2012).

## Results

In the multilevel CFA, we examined our hypothesized four-factor measurement model in which we included our level 1 variables (i.e., workload, detachment, and marital satisfaction) at the within-person level and our level 2 variable (i.e., work-home segmentation preference) at the between-person level. Overall, this model had a good fit with the data (CFI = 0.96, TLI = 0.93, RMSEA = 0.07, and SRMR = 0.06; Kline, 2005). In addition, each item loaded significantly and in the expected direction onto its respective latent factor. Moreover, our hypothesized measurement model yielded superior fit compared to different alternative models (results available by request from first author). Combined, these research findings support the distinctiveness of our study constructs.

### Descriptive statistics

Table 1 reports the means, standard deviations, intraclass correlations, zero-order correlations (i.e., the correlation at the individual level) and person-centered correlations (i.e., the correlation at the day-level). On average, our respondents rated their daily level of experienced workload with a 3.11 out of 5, suggesting they perceived themselves as working in a demanding work environment. This result aligns with findings from a recent study that examined workload in the Flemish workforce (i.e., our sample background; Bourdeaud'hui and Vanderhaeghe, 2013). As all intraclass correlation coefficients at the day-level were higher than 0.05—indicating a considerable amount of the variability in these variables is due to within-person differences (Marcoulides and Schumacker, 2009)—we are confident that the variables fluctuated over time. Specifically, about 50% of the variation in each of our level 1 variables [i.e., workload (47%), detachment (45%) and marital satisfaction (54%)] was due to day-to-day fluctuations. Note that we only report the correlations at the zero-order level for work-home segmentation preference as this variable was only measured at the between-person level and thus renders the estimation of within-person correlation obsolete.

**Table 1:** **Means, standard deviations, intraclass correlations, zero-order, and person-centered correlations among the focal variables**.

	***M***	***SD***	**ICC (person)**	**ICC (day)**	**1**.	**2**.	**3**.	**4**.	**5**.
1. Workload	3.11	0.96	0.53	0.47		−0.03	−0.31^***^	−0.16^***^	−
2. Workload squared	10.51	5.90	0.53	0.47	−0.2		−0.04	0.01	−
3. Detachment	3.45	1.09	0.55	0.45	−0.05	−0.02		0.22^***^	−
4. Marital satisfaction	4.22	0.79	0.46	0.54	−0.08^**^	−0.06	0.01		−
5. Work-home segmentation preference	3.58	0.87	1.00	-	0.06^*^	0.06^*^	0.03	−0.07^*^	

* *p < 0.05*.

** *p < 0.01*.

*** *p < 0.001. Means and standard deviations were computed on the raw data. Zero-order correlations are presented below the diagonal (N = 136). Person-centered correlations are presented above the diagonal (N = 1144)*.

### Hypothesis testing

**Figure 2:**
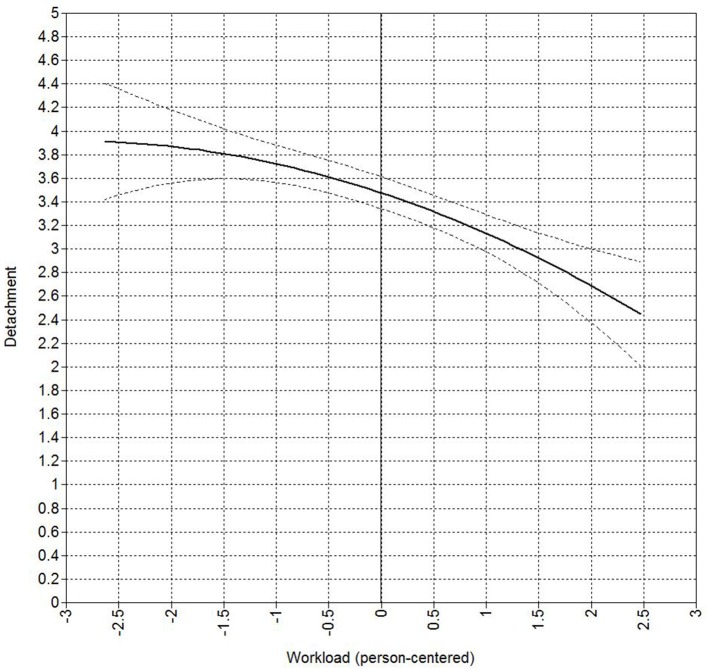
**Curvilinear relationship between workload and detachment on a daily basis**. The black solid line represents the curvilinear relationship, whereas the black dotted lines represent a 95% credibility interval.

We tested a mediation model in which detachment was predicted by the linear and squared effect of workload and marital satisfaction was predicted by the linear effect of detachment while controlling for the direct effect of the linear and squared effect of workload on marital satisfaction. In addition, the mediation model contained the random slope of detachment on marital satisfaction that was regressed on an employee's preference to segment work and home domains. Our results indicate a negative linear (95% CI for θ = −0.37 is −0.44 to −0.30) as well as squared (95% CI for θ = −0.07 is −0.13 to −0.01) effect of workload on detachment on a daily-level, thereby supporting hypothesis 1. Figure 2 represents the curvilinear relationship between workload and detachment. As can be seen in Figure 2, between (very) low and average levels of workload the negative impact on detachment is small and not significantly different, however as from the average level of workload every increase in workload leads to a stronger decrease in detachment.

Furthermore, we found a direct positive effect of detachment on marital satisfaction (95% CI for θ = 0.14 is 9.09 to 0.18), thereby supporting hypothesis 2. We found a direct significant negative effect of linear workload on marital satisfaction (i.e., not mediated by detachment; 95% CI for θ = −0.07 is −0.12 to −0.02), whereas our results did not indicate a significant relation between the squared effect of workload and marital satisfaction (95% CI for θ = 0.01 is −0.03 to 0.05) on a daily-level.

**Figure 3:**
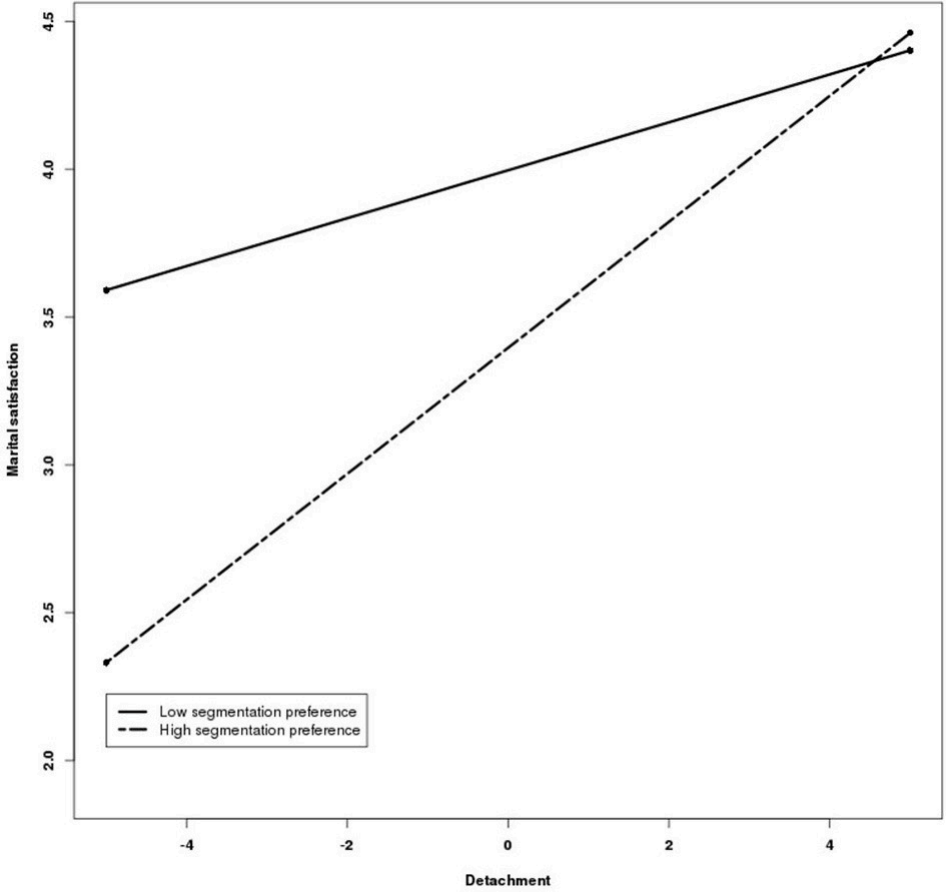
**Cross-level interaction of work-home segmentation preference**.

In line with hypothesis 3, work-home segmentation preference positively moderated the relationship between detachment and marital satisfaction (95% CI for θ = 0.07 is 0.03 to 0.12). Put differently, the positive relationship between detachment and marital satisfaction is stronger among employees who prefer to segment work from home. Figure 3 represents the results of the moderated two-level path analysis used to test the third hypothesis. However, preferring to keep work and home segregated from each other has a direct negative effect on marital satisfaction (95% CI for θ = −0.33 is −0.52 to −0.15).

**Figure 4:**
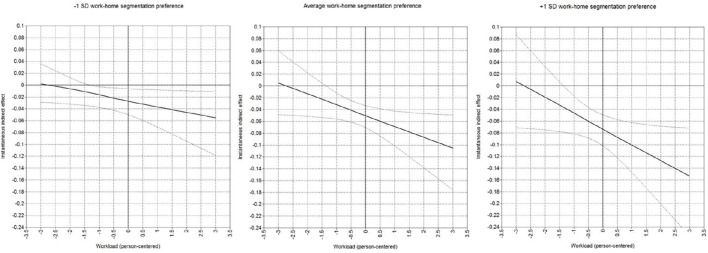
**The instantaneous indirect mediation effect of workload on marital satisfaction via detachment as a function of person-centered workload**. The mediation effect is depicted for employees who scored 1 standard deviation below the average (left), average (middle), and 1 standard deviation above the average (right) on work-home segmentation preference. The black solid line represents the mediation effect, whereas the black dotted lines represent a 95% credibility interval.

The mediation effect specified in hypothesis 4, contains a curvilinear relationship between workload and detachment, as well as a linear relationship between detachment and marital satisfaction. To that end, we relied on the approach specifically developed to address non-linear mediation (Hayes and Preacher, 2010). As such, we evaluated the indirect non-linear mediation effect of workload on marital satisfaction via detachment for different values of workload (i.e., instantaneous indirect effect). The mediation effect does not only depend on the curvilinear relationship between workload and detachment, and the linear relationship between detachment and marital satisfaction, but is also conditional on the level of workload. To that end, the instantaneous indirect effect was tested for the average value of workload and for two and one standard deviation(s) below and above the average value of workload. Daily detachment fully mediated the relationship between workload and marital satisfaction for every predefined level of workload, thereby supporting hypothesis 4. We found that the instantaneous indirect effect through detachment is negative and significant when workload is two (95% CI for θ = −0.03 is −0.05 to −0.00) and one (95% CI for θ = −0.04 is −0.06 to −0.02) standard deviations below the mean, when workload is average (95% CI for θ = −0.05 is −0.07 to −0.03) as well as when workload is one (95% CI for θ = −0.06 is −0.09 to −0.04) and two (95% CI for θ = −0.07 is −0.11 to −0.04) standard deviations above the mean, for employees scoring average on work-home segmentation preference. This mediation effect is depicted in Figure 4 together with the 95% credibility intervals for employees with low (−1 *SD*), average and high (+1 *SD*) levels of work-home segmentation preference. Irrespective of the level of work-home segmentation preference, the instantaneous indirect effect is positive for very low levels of workload (i.e., −3 *SD*). In other words, at very low levels of workload, increases in workload will be beneficial for an employee's marital satisfaction via workload's influence on detachment. However, the instantaneous indirect effect of workload on marital satisfaction via detachment is negative for low (i.e., −2.5 *SD*) up to very high (i.e., +3 *SD*) levels of workload, irrespective of the level of work-home segmentation preference. In other words, further increases in workload lowers marital satisfaction via the negative effect of workload on detachment. The negative influence of workload on marital satisfaction via detachment becomes stronger (i.e., accelerating decreasing curve) for employees with a high (+1 *SD*) compared to low (i.e., −1 *SD*) work-home segmentation preference.

## Discussion

We demonstrated the existence of a curvilinear relationship—on top of the already acknowledged linear relationship—between workload and psychological detachment and a linear relationship between detachment and marital satisfaction, before examining and illustrating the mediating role of detachment—as defined in the stressor-detachment model—in the daily relationship between workload and marital satisfaction. Furthermore, our findings show that an employee's stable preference to segment the work and home domain strengthens the daily relationship between detachment and marital satisfaction. In doing so, our study contributes to the stressor-detachment and work-home interface literature.

### Discussing the results, their implications, and alternative explanations

We found support for a negative linear as well as curvilinear relationship between workload and detachment. The linear relationship between workload and detachment denotes the general negative trend that was often illustrated in previous studies, being the more workload experienced at work, the less an employee will be able to detach that day (Sonnentag and Bayer, 2005). In addition, the negative curvilinear relationship provides a more nuanced picture of the relationship between workload and detachment. As the curvilinear relationship did not display the hypothesized inverted U-shape, there is no optimal moderate level of workload which allows employees to detach better. Instead, we found that an equal increase in levels of workload did not lead to an equal decrease in the level of detachment. Specifically, from very low till moderate levels of workload, the increase in negative effects for detachment is not prominent. As such, our results suggest that the decline in the ability to detach is negligibly small in case an employee experiences very low, low or moderate levels of workload during his/her workday. However, once workload reaches a moderate level every further increase is likely to result in an accelerating decline of the ability to detach from work. In other words, whereas small increases in workload over workdays do not alter the ability to detach substantially when the experienced levels of workload are very low to moderate, every small increase in workload above moderate levels will further dwindle an employee's ability to detach from work substantially. Combining our results suggest that every increase in workload reduces an employee's ability to detach from work, with particularly detrimental effects for detachment once workload exceeds moderate levels. Although we hypothesized that the evoked stress of underload and overload would interfere with detachment (Sluiter et al., 2003; Sonnentag et al., 2010a), our research finding could be framed in the light of conservation of resources theory if we argue that detachment will be less likely in the absence of resources (Hobfoll, 1988, 1989). Very low to moderate levels of workload increasingly call upon resources, however the additional resources needed to handle these levels of workload seem to be manageable and as such only slightly interfere with detachment. However, confronted with moderate to very high levels of workload, an employee will increasingly need to consume resources to be able to cope with the workload at hand, possibly explaining the exponential negative effect on detachment. However, another alternative explanation for the absence of the hypothesized inverted U-shaped relationship between workload and detachment could be our focus on detachment during off-job time. Prior research indicated that detachment can take place after as well as during working time (Trougakos et al., 2008). Detaching at work could be especially relevant for employees who experience low levels of workload and potentially have more time available to devote to detachment activities during workhours^1^. As such, it could be valuable to examine how the curvilinear relationship between workload and detachment evolves and develops within one workday by assessing respondents' workload and detachment multiple times a day (e.g., by using an experience sampling design with multiple short questionnaires during the workday as well as at home). This would allow scholars to examine the influence of different levels of workload on detachment during off-job hours, while controlling for detachment during workhours as well as on the (combined) act of detaching during and after workhours. Nevertheless, our research finding opens up new avenues to examine potential non-linear relationships between job demands and one's ability to detach on a daily basis.

In addition, detachment positively influenced feelings of marital satisfaction. This effect could be ascribed to the resource replenishing and further resource taxation inhibiting characteristics of detachment. These replenished, available resources are necessary in order to engage in positive relational functioning, communication and behavior (Neff and Karney, 2009; Randall and Bodenmann, 2009). In line with the conservation of resources theory, detachment might conserve, protect resources and prevent further resource loss, which will positively impact an employee's feelings of marital satisfaction (Hobfoll, 1988).

Furthermore, an employee's stable preference to keep his/her work and home domain separated will enhance the positive influence of detachment on marital satisfaction. This finding aligns with person-environment fit theories that predict positive outcomes when an employee's behavior (i.e., ability to detach from work) and preference (i.e., work-home segmentation) are in line (Kristof-Brown et al., 2005). Alternatively, employees with a preference to segment work from home who are not able to detach can possibly experience boundary violation which will result in negative home outcomes (i.e., marital dissatisfaction; Kreiner et al., 2009). In addition, we found a negative effect of preferring to keep work and home life separated from each other on an employee's marital satisfaction. One possible explanation for this somewhat surprising finding could be that some of the individuals who like to segment work from home communicate less about work with their partner who's willing to talk about work. Another possible explanation could be that the partner who prefers to segment work from home is also less willing to listen when his/her partner wants to talk about work-related matters and as such feels less supported by his/her partner. It could be valuable to examine whether this negative effect between preferring to segment work from home and marital satisfaction is influenced by the (dis)congruency in work-home segmentation preferences between partners.

Lastly, our results suggest an indirect effect of workload on marital satisfaction through an employee's ability to detach from work. In other words, we found that the negative influence of workload on marital satisfaction operates through the negative effect on detachment. This depleting effect becomes stronger when the initial level of workload increases as well as with an increase in employee's stable work-home segmentation preference. This finding is in line with the stressor-detachment model that states that detachment exerts a mediating role between work stressors and strain reactions (Sonnentag and Fritz, 2015). In addition, our study provides further evidence to examine the stressor-detachment model on a daily basis as about 50% of the variance in workload, detachment and marital satisfaction was located on the day-level.

### Limitations

Notwithstanding the methodological and theoretical contributions of our study, we need to acknowledge some limitations. First, we assessed our variables with self-report measures, which might raise concerns about social desirability and common method variance (Podsakoff et al., 2003). However, we eliminated between-person variance and variance caused by individual response tendencies by person-mean centering the variables (Ilies et al., 2010) and since common method variance cannot explain nor alter interaction effects (i.e., significant cross-level interaction with work-home segmentation preference; Siemsen et al., 2010), we assume these biases only scarcely influenced our results. Moreover, to check for common method variance, we performed a Harman's single-factor test (Podsakoff et al., 2003; Krishnaveni and Deepa, 2013). This test examines whether the data provides a good fit with a one-factor model, which would suggest one underlying latent factor due to common method variance. However, the fit indices of the exploratory factor analysis were not satisfactory (see Appendix B in Supplementary Material for the test results). The absence of one common underlying latent factor is also supported by several small to zero correlations between our study variables, both at the within- and the between-person level (see Demerouti et al., 2007 for a similar reasoning). Combining the abovementioned arguments, we assume it is very unlikely that common method variance had a major impact on our results. Nevertheless, we advise further research to include other-ratings of workload in the light of the current organizational trend to rely on teamwork (e.g., direct colleague and/or supervisor reports; Ilgen and Pulakos, 1999). In addition, future research would benefit from the use of objective workload indicators such as amount of attained objectives, for example the number of outgoing phone calls for call center employees. Another way to objectify the measure of workload could be by relying on physiological measures such as heart rate variability. Moreover, the possibility of common method variance could be reduced even more by measuring the predictor and outcome variables separated in time, such as across two daily diary surveys (i.e., experience sampling design) were employees are instructed to fill out their experienced levels of workload at the end of the workday and the experienced levels of detachment and marital satisfaction right before bedtime (Podsakoff et al., 2003).

Second, we used self-reported time stamps in our paper- and pencil surveys. Studies examining the work-home interface often rely on paper- and pencil booklets to avoid attrition due to assessing variables at work as well as at home (for a similar approach see Volman et al., 2013). Although we chose this approach to allow respondents without work laptop or internet access at home to participate in the study and to avoid respondents to check their (potential work-related) emails late in the evening, we cannot verify the truthfulness of their indicated time stamps. However, we took some steps to minimize the potential that respondents would untruthful indicate time stamps. That is, we instructed our respondents to leave the survey blank, instead of filling them out later that day or on the next day, in case they forgot to fill it out. As some respondents left some surveys blank while they did indicate that they went to work that day, we are relatively confident that the self-reported time stamps are trustworthy. Moreover, participation was strictly voluntary with no incentive contingent on completion of the surveys. Hence, respondents have little external motivation to retrospectively complete the surveys. However, to objectify the time and day of survey completion, we recommend future research to rely on electronic surveys with automatic time stamps.

Third, the impact of surveying employees in itself on their detachment level that day remains unknown^1^. In our study, we assessed all variables during an employee's private time. However, the act of completing the questionnaire might provoke work-related thoughts. To restrict the potential negative effect, we asked them to fill out the booklet right before going to bed. As such, we did not induce work-related thoughts between the time of arriving home and going to bed. Moreover, we instructed all respondents at the start of the study that they could withdraw at any time. In addition, when collecting the booklets, we checked whether the respondents had encountered any harm by completing the booklet. However, it would be a valuable area for future studies to examine whether the act of completing daily surveys concerning work- and home-related behavior, emotions and cognitions in itself impacts the respondent's well-being.

Lastly, we recruited respondents by means of a convenience sampling design, which potentially resulted in a sample that is not representative of the general population. However, recent meta-analytic findings (Wheeler et al., 2014) suggest slightly lower effect sizes and correlations in convenience samples compared to non-convenience samples, whereas the same overall conclusions could be drawn from both samples. Hence, the use of a convenience sample would have resulted in more conservative estimates of the relationships between the variables under study. In addition, convenience samples are less problematic in within-person studies since employees are compared with themselves rather than to others (see Debusscher et al., in press for a similar reasoning).

### Suggestions for future research

The current study opens up new avenues for further research. Besides looking at the individual's within-person relationships, another direction for future research could be to look into crossover effects (i.e., transference of an individual's effect on another individual). An employee's experience of workload could depend on and/or influence the performance within his/her work group (i.e., between colleagues) and impact colleagues' ability to detach from work during job breaks. In a similar vein, within dual-earner couples (i.e., between partners) the workload an employee experiences at work may influence his/her partner's ability to detach from his/her work at home.

Furthermore, it would be valuable to find buffering effects for the negative influence of workload on detachment from work. Previous between-person studies found support for a buffering effect of social support on the relationship between workload and stress (Glaser et al., 1999). It is hence recommended for future research to investigate whether the hindering effect of workload on an employee's ability to detach, is diminished by having resources at his/her disposition on a daily basis.

Lastly, we extended the stressor-detachment model, by finding support for a curvilinear—on top of the frequently examined linear—effect of workload on detachment and empirically demonstrating the mediating role of detachment. It is worth mentioning that we operationalized workload in our study as quantitative workload (i.e., too many tasks to handle in too little time), whereas future studies could examine the effects of qualitative workload (i.e., too difficult/complex tasks to handle). In addition, we recommend to broaden the scope of the stressor-detachment model to also include the possibility of curvilinear relationships. For instance, the cognitive demands an employee experiences at work can have negative (too little or too much cognitive demands) as well as positive (moderate amount of cognitive demands) consequences for an employee's capacity to detach form work. In addition, more research needs to be done, to examine the nature of the mediation effect of detachment in the relationship between job stressors and relational, well-being and home outcomes.

### Practical implications

Given the importance of workload in today's work environment, understanding the temporal relationship with detachment and marital satisfaction provides policy makers with a powerful instrument.

Firstly, it is important to raise employees' awareness of the potential consequences of having to manage too much work. Our study highlights that encountering workload does not interrupt with the ability to detach from work substantially as long as the workload is not too high (i.e., exceeding moderate levels of workload). We emphasize that it's important for an employee to recognize the necessity to set boundaries and discuss them with the supervisor to prevent a high workload from damaging his/her family life by diminishing the capacity to mentally let go of work. Moreover, it's worthwhile to foster supervisors' acknowledgment to lead by example; that is managers should aim to handle work in a way that the workload remains manageable.

Secondly, detachment mediates the relationship between workload and marital satisfaction. In this respect, it is important to mention that the capacity to “mentally switch off” from work can be trained (Hahn et al., 2011). This training would be particularly interesting for employees who experience high workload at work as well as individuals who prefer to integrate work and life domains.

Thirdly, it is important that employees are aware of their own preference to segment or to integrate the work and home domain. Moreover, they should try to act congruent with their preference, recognize the preference of others and try to respect this preference and behave accordingly.

## Ethics statement

The Ethical Commission Human Sciences (Ethische Commissie Humane Wetenschappen) of the Vrije Universiteit Brussel granted ethical approval for the study (reference number ECHW_045).

## Author contributions

LG and SD exchanged research ideas and designed the outline of the article together during several meetings. Afterwards, LG and SD each collected data based on the surveys they developed together. LG did the analyses and interpreted the results. LG drafted the article and SD revised/edited the article. LG and SD both made substantial contributions to the work reported in the article. LG will act as a corresponding author for the article.

## Funding

This work was supported by grants from the Research Foundation Flanders, Belgium (FWO-Vlaanderen) awarded to LG (grant number 11Q6414N). All authors are independent of their funders.

### Conflict of interest statement

The authors declare that the research was conducted in the absence of any commercial or financial relationships that could be construed as a potential conflict of interest.

## Footnotes

^1^We thank the reviewer for this suggestion.
