# Supplementary material for: Psychological Detachment Mediating the Daily Relationship between Workload and Marital Satisfaction
Source: Front Psychol. 2017 Jan 4;7:2036. doi: 10.3389/fpsyg.2016.02036 (PMC5209365; doi:10.3389/fpsyg.2016.02036)
Supplement: Supplementary file 1 [file DataSheet1.docx]

**Appendix A**

We examined boundary conditions of our hypothesized relationships. To rule out possible inequivalent effects of full-time versus part-time working, we examined whether the strength of our hypothesized relationships significantly differed between full-time and part-time workers (i.e., multi-group equivalence test; Byrne & van de Vijver, 2010). One could for example argue that part-time workers can devote more time on leisure activities and as such would be better able to detach compared to full-time workers. Specifically, we compared (1) the configural model in which we imposed no equality constraints (i.e., regression parameters estimations are working time specific) and (2) an equality constrained model (i.e., regression parameters estimations are imposed to be equal for (no) full-time workers; Byrne, Shavelson, & Muthén, 1989). We conducted a likelihood-ratio test to compare both models since the equality-constrained model is nested within the configural model. Based on the insignificant result of the test (χ^2^(4, *N* = 136) = 2.50, *p* =.65) we conclude that the relationships between the focal variables did not differ between full-time and part-time workers.

**Appendix B**

We conducted a Harman’s single-factor test to examine whether the different items and scales in our study can be represented by a single common method factor. We specified one factor at the within-person level and saturated the between-person level. The results of the exploratory factor analysis indicated a bad fit between the one-factor model and the data (χ²(27) = 1015.31; *p* < .001; *CFI* = .71; *TLI* = .22; *RMSEA* = .11; *SRMR_(within)_* = 0.13). Hence, Harman’s single-factor test suggests that there is no strong methods factor in the data.
